# Clinical validation of urinary indole-reacted calcium oxalate crystallization index (iCOCI) test for diagnosing calcium oxalate urolithiasis

**DOI:** 10.1038/s41598-020-65244-1

**Published:** 2020-05-20

**Authors:** Pimkanya More-krong, Praween Tubsaeng, Natcha Madared, Monpichar Srisa-Art, Numpon Insin, Pannee Leeladee, Chanchai Boonla

**Affiliations:** 1 Department of Biochemistry, Faculty of Medicine, Chulalongkorn University, Bangkok, 10330 Thailand; 2 Division of Urology, Mahasarakham Hospital, Mahasarakham Province, 44000 Thailand; 3 Department of Chemistry, Faculty of Science, Chulalongkorn University, Bangkok, 10330 Thailand

**Keywords:** Diagnostic markers, Urology

## Abstract

An indole-reacted calcium oxalate crystallization index (iCOCI) test was developed to quantify the total competence of urine to precipitate calcium oxalate (CaOx) crystals. We conducted the prospective cohort study in accordance with the STARD guideline to evaluate the accuracy of urinary iCOCI test (index test) for diagnosing urolithiasis. A total of 281 participants were recruited for the study. Levels of urinary iCOCI were determined in the pre-diagnostic 24-h urine samples. Positive urinary iCOCI (≥ 0.6 COM eqv., g/L) was accounted for 51% (144/281), and the rest of 49% (137/281) were negative. Non-contrast CT imaging (reference standard) was subsequently performed for the definite diagnosis of urolithiasis to divide the participants into two groups, non-stone subjects (NSS, n = 122) and stone-forming subjects (SFS, n = 159). It should be noted that only subjects who currently had urinary stone at the time of study were classified as SFS. Urinary iCOCI levels in the SFS were significantly higher than the NSS. ROC analysis revealed an area under curve (AUC) of 0.893 (95% CI: 0.855–0.932) in separating NSS from all SFS. Sensitivity, specificity, positive predictive value (PPV), negative predictive value (NPV), accuracy, positive likelihood ratio (LH^+^) and negative likelihood ratio (LH^−^) of urinary iCOCI test for diagnosis of all urolithiasis were 87%, 80%, 84%, 84%, 83%, 4.44 and 0.16, respectively. Of 159 SFS, 38 were confirmed to have CaOx stones. Among these 38 CaOx SFS, only 2 had negative urinary iCOCI test. The AUC of urinary iCOCI test for separating CaOx SFS from NSS was markedly high (0.946, 95% CI: 0.914–0.978). Sensitivity, specificity, PPV, NPV, accuracy, LH^+^ and LH^−^ of urinary iCOCI test for diagnosing CaOx urolithiasis were 95%, 86%, 68%, 98%, 88%, 6.80 and 0.06, respectively. Conclusion, we clinically validated that an innovative non-invasive urinary iCOCI test was highly accurate to diagnose urolithiasis, especially CaOx stone. With its high sensitivity and NPV, urinary iCOCI test is clinically intended to use as a screening test for CaOx urolithiasis. LH^−^ of 0.06 indicates that negative result of urinary iCOCI test is highly accurate to rule out the CaOx stone formation. It is noted that urinary iCOCI level is expressed as arbitrary unit, and it is not directly related to the actual physiological level of urinary oxalate.

## Introduction

Oxalate is toxic to human. It is endogenously synthesized in the liver from glycolate and glyoxylate and excreted to urine by kidneys. Oxalate is also exogenously derived from diets such as edible plants, vegetables, fruits, nuts and grains^1^. Acute toxicity of oxalate is mainly caused by ethylene glycol poisoning^2^. In contrast, chronic toxicity of oxalate is primarily due to persistently high urinary excretion of oxalate that leads to calcium oxalate (CaOx) crystallization and urinary stone formation^3^. Urinary stone disease or urolithiasis is a common urological problem worldwide, particularly in the tropics. The most common type of urinary stones is CaOx, found up to 90% varying among countries^4–8^. Thus, measurements of urinary oxalate and capability of CaOx crystallization in urine are important for estimating the risk of CaOx urolithiasis development.

Laube *et al*. established the first measurement of urinary CaOx crystallization index for estimating the actual risk of CaOx formation, called Bonn Risk Index (BRI)^9^. BRI is calculated from urinary ionized calcium concentration divided by amount of ammonium oxalate added to urine to induce CaOx crystallization. Clinical usefulness of BRI was evaluated and compared with other stone risk evaluation methods, *i.e*., CaOx supersaturation (RS_CaOx_) and CaOx activity product (AP_CaOx_), revealing that BRI had the highest reliability in distinguishing healthy subjects from CaOx stone formers. Based on receiver operating characteristic (ROC) analysis, BRI (80% sensitivity, 70% specificity) had higher area under ROC curve (AUC) than RS_CaOx_ (65% sensitivity, 78% specificity) and AP_CaOx_ (55% sensitivity, 87% specificity), respectively^10^. Recently, we developed a novel test for quantifying ability of urine to perform CaOx crystallization, called calcium oxalate crystallization index (COCI) test^11^. The urinary COCI test has high discriminatory power (83% sensitivity, 97% specificity, 90% accuracy) in separating stone-forming subjects (SFS) from the age- and sex-matched non-stone subjects (NSS) with an AUC of 0.9499 (95%CI: 0.9131–0.9868). With this diagnostic accuracy, the urinary COCI test is promising for clinical implementation. However, in our previous study we did a cross-sectional case-control study, and we measured urinary COCI in the post-diagnosis 24-h urine specimens. Therefore, biases of participant recruitment and disease spectrum could be introduced. The other problem was that the crystals obtained from the COCI procedure (so-called COCI crystals) were not solely CaOx crystals. Other crystals, mainly calcium phosphate (CaP) crystals, were also produced. We quantified the amount of the produced COCI crystals by measuring absorbance at 215 nm, which was not exclusively specific to oxalate. To measure only the CaOx crystals, a chemical reaction that selectively or specifically reacts with oxalate must be incorporated into the COCI procedure. Bergerman and Elliot in 1955 reported a specific direct reaction of oxalate with indole to produce a pink-colored compound^12^. Later, the indole reaction was demonstrated by Hausman *et al*. in 1956 for measurement of oxalate in urine samples^13^. Therefore, we incorporated this indole-oxalate reaction into the original COCI test in order to detect only the CaOx crystals produced from the COCI procedure and named it as indole-reacted COCI or iCOCI test.

The purpose of the present study was to develop a urinary iCOCI test and evaluate its diagnostic accuracy in separating NSS from SFS, especially from CaOx SFS. It is well-accepted that the diagnostic accuracy study has a higher risk of bias than other clinical studies. Therefore, we complied with the STARD guideline for reporting the diagnostic accuracy of the index test (urinary iCOCI test) compared against the reference standards (CT scan for all stones, FTIR for CaOx stone)^14,15^. The clinically intended use of urinary iCOCI test is to be a screening test or a medical surveillance for assessing the likelihood of having urinary stone formation, particularly CaOx stone (similar to cholesterol measurement for assessing the risk of heart disease), in the defined population who have higher risk of stone formation than normal, e.g., individuals who have positive family history of urolithiasis^16^, men aged over 30 years old^17^ and women after menopause^18^. The clinical role of this newly developed urinary iCOCI test is proposed to be a non-invasive triage test to identify peoples at risk of having CaOx stone formation before performing CT scan or other diagnostic imaging modalities. Since a screening test does not function to diagnose the disease, and individuals with screening test positive subsequently require further testing with a diagnostic test for the disease confirmation^19^, we believe that the use of urinary iCOCI test would significantly reduce the number of individuals requiring CT scan for diagnosis of urinary stones, and hence drastically reducing the imaging cost and radiation exposure.

## Subjects and Methods

### Participants and specimen collection

To minimize the spectrum bias of this diagnostic accuracy study, the urinary iCOCI levels were determined in pre-diagnostic 24-h urine samples^20^. Thailand is endemic for urinary stone disease, especially in the rural villages of the northeastern region. Therefore, two study sites were set in a northeastern province, Mahasarakham Province. One site was in the Kaedam District consisting of 13 rural villages, and the other was the provincial hospital of Mahasarakham. Potentially eligible participants were rural villagers in the Kaedam District (n = 139), and patients came to see the doctors and admitted to the urology ward of Mahasarakham hospital (n = 143), aged ≥ 18 years old and both men and women. All participants gave informed consents before recruitment and specimen collection. Research protocol was reviewed and approved by the Ethic Committee of Faculty of Medicine, Chulalongkorn University, and Ethic Committee of Mahasarakham hospital. All methods were performed in accordance with the relevant guidelines and regulations.

**Figure 1:**
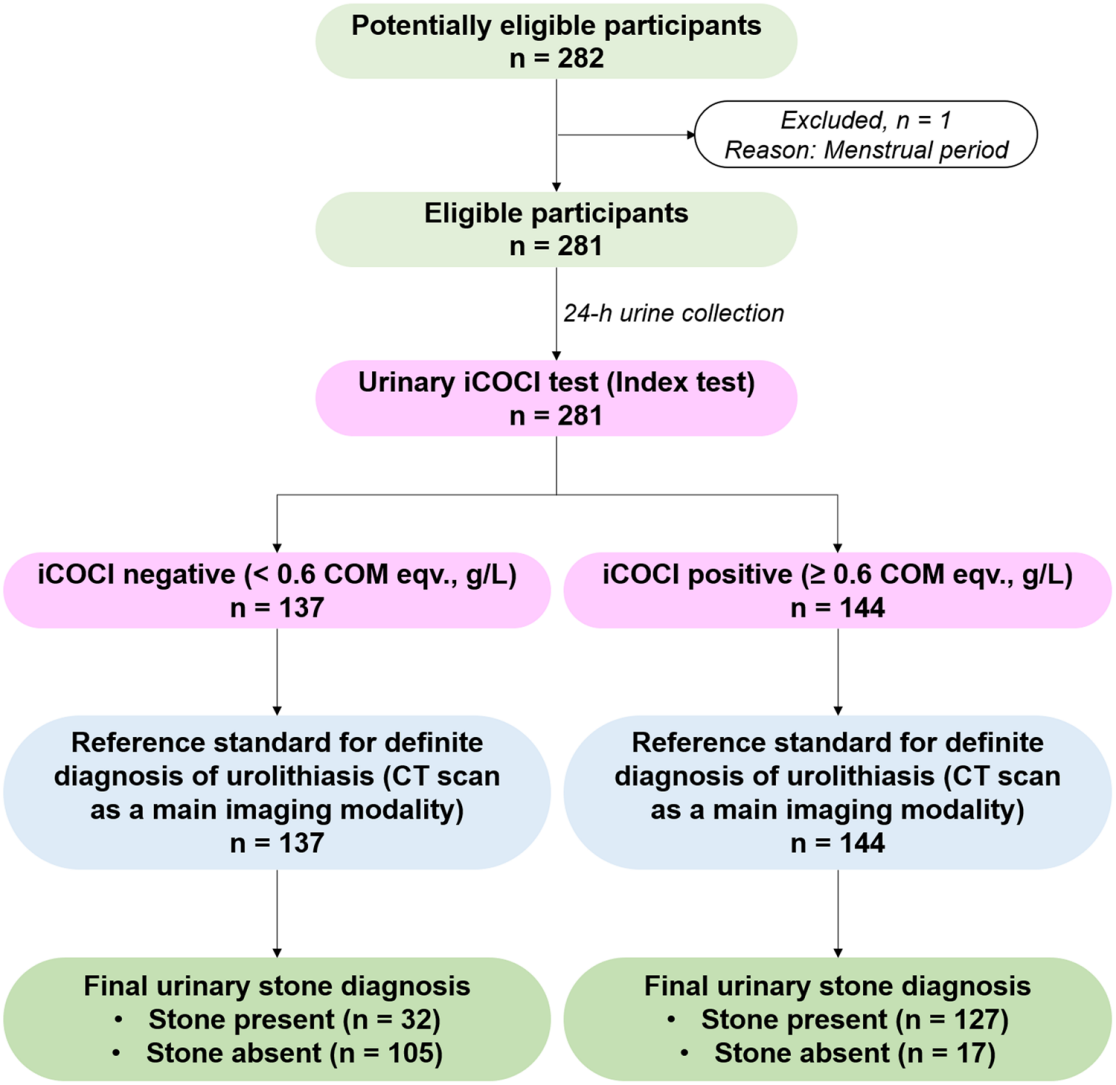
STARD flow diagram of the study for all stone cases (n = 159).

A total of 282 participants were potentially eligible for the study (Fig. 1). One participant from the village was excluded because she had a menstrual period on the day of recruitment. Therefore, 281 eligible participants were recruited and instructed for 24-h urine collection. All of 138 recruited villagers were strictly instructed to collect 24-h urine specimens by themselves at home. One hundred and forty-three recruited patients collected their 24-h urine specimens in the hospital (facilitated by nurses). During the urine collection, all participants were asked to avoid oxalate-rich vegetables and not to drink coffee and tea. The iCOCI test was performed in 281 urine samples. Non-contrast CT scan was employed as a main reference standard for definite diagnosis of urolithiasis (evaluated and interpreted by a radiologist of Mahasarakham hospital). In cases with inconclusive result of CT imaging, other imaging modalities (kidney, ureters, bladder (KUB) X-ray and ultrasound imaging) together with symptoms related to stone disease were taken into consideration for the definite diagnosis of urolithiasis (by urologist). The cutoff value for urinary iCOCI test was set at 0.6 calcium oxalate monohydrate equivalent (COM eqv.), g/L. Urinary iCOCI values of < 0.6 were labeled as negative. The values of ≥ 0.6 COM eqv., g/L were iCOCI positive. A total of 122 subjects were absent for urinary stone and classified as NSS. One hundred and fifty-nine participants were present with urinary calculi and classified as SFS. Of 159 SFS, 16 were participants from the villages, and the rest were from the hospital. Basically, majority (up to 80–90%) of the patients admitted to the urology wards in the northeastern hospitals of Thailand were urolithiasis patients (personal communication with the local urologists). In this study, all 143 voluntarily participated patients (100%) from the urology ward were diagnosed with urolithiasis. Of 143, 140 patients had clearly positive CT scan for urinary stones. Three cases had negative (inconclusive) CT scan results, but they had positive KUB X-ray and ultrasound imaging with clear symptoms of urolithiasis. Therefore, the urologist decided to treat them as urolithiasis patients, and we classified them as SFS for further analysis.

Demographic and characteristics of NSS and SFS groups are shown in Table 1. Of 159 SFS, 33 (20.75%) had recurrent stones (all from the hospital), and 69 had stone specimens available for stone composition analysis by Fourier-transform infrared spectroscopy (FTIR). Stone types were categorized into three main groups according to the major mineral component, *i.e*., CaOx (n = 38), CaP (n = 19) and uric acid (UA, n = 12) stones (Supplementary Fig. 1 for FTIR spectrum of each stone type).

**Table 1:** Demographic and characteristics of NSS and SFS groups.

Characteristics	NSS	SFS
Number of subjects	122	159
Age (years)		
• Mean ± SD	50.68 ± 9.43	56.66 ± 10.72
• Median (IQR)	50 (13)	55 (14)
Sex		
• Male (%)	38 (31)	101 (64)
• Female (%)	84 (69)	58 (36)
BMI (kg/m^2^, n = 273)		
• Mean ± SD	24.32 ± 5.36	23.83 ± 4.90
• Median (IQR)	24.80 (5.35)	23.88 (5.73)
Urine volume (mL)		
• Mean ± SD	1327 ± 641	1419 ± 604
• Median (IQR)	1160 (805)	1350 (970)
Urine pH		
• Mean ± SD	6.63 ± 0.76	6.98 ± 1.00
• Median (IQR)	6.59 (0.91)	6.89 (1.29)
Urine creatinine (g/day)		
• Mean ± SD	2.12 ± 0.80	1.60 ± 1.39
• Median (IQR)	2.00 (0.95)	1.24 (1.34)
Urinalysis (by urine strip)		
Ketone (%)		
• Negative	118 (96.7)	157 (98.7)
• Positive	4 (3.3)	2 (1.3)
Glucose (mg%, %)		
• Negative	108 (88.5)	144 (90.7)
• ≥250	6 (4.9)	12 (7.5)
• 500	7 (5.7)	2 (1.3)
• 2000	1 (0.9)	1 (0.5)
Protein (mg/L, %)		
• Negative	108 (89)	142 (89)
• ≤150	11 (9)	13 (8)
• >150	3 (2)	4 (3)
Blood (%)		
• Negative	122 (100)	154 (97)
• Positive	0	5 (3)
Stone types by FTIR (n = 69)		
• Calcium oxalate		38 (55.07)
• Calcium phosphate		19 (27.54)
• Uric acid		12 (17.39)

NSS: non-stone subjects, SFS: stone-forming subjects, SD: standard deviation, IQR: interquartile range, FTIR: Fourier-transform infrared spectroscopy.

### Urinary iCOCI test

Urine samples were centrifuged at 4,000 rpm for 5 min. Supernatant was collected and filtered through 0.22 μm membrane for iCOCI testing. Various concentrations of COM solution were prepared (0.093, 0.163, 0.375, 0.75 and 1.50 g/L) in 2 N HCl for creating COM standard curve. The iCOCI measurement was performed based on the original COCI test with minor modifications, as followed^11^. The filtered urine sample (950 μL) was spiked with oxalate (50 μL of 80 mM oxalic acid) to achieve final concentration of 2 mM oxalate. Oxalic acid solution (2 mM final concentration) was prepared (50 μL of 80 mM oxalic acid added to 950 μL of water) to be used for subtraction of absorbance from the oxalate-spiked urine. CaCl_2_ solution (100 mM, 1 mL) was added to the oxalate-spiked urine and the oxalic acid solution (final volume of 2 mL), mixed well and then incubated at 37 °C for 10 min for crystallization. The crystals generated, called COCI crystals, were harvested by centrifuge at 15,000 rpm for 15 min. The COCI crystals were washed once with distilled water. After centrifuged (15,000 rpm for 15 min), the COCI crystals were collected and dried at room temperature for 10 min. The crystals were solubilized by 2 N HCl (500 μL) for further indole reaction.

Indole reagent (1 mg/mL) was freshly prepared in concentrated H_2_SO_4_. The COCI crystal solution (250 μL) was mixed 1:1 with indole reagent (250 μL). The mixture was incubated at 80 °C for 45 min. Oxalate directly reacted with indole to yield the pink-colored product. The color intensity was proportional to the concentration of oxalate. After cooled, absorbance at 530 nm was measured (iCOCI absorbance). Indole reaction of the standard COM solution was carried out in the same way for generating the standard curve. To obtain the real iCOCI absorbance of the original urine (before spiking), iCOCI absorbance of the oxalate-spiked urine was subtracted from the absorbance of 2 mM oxalic acid solution. The real iCOCI absorbance was used to calculate amount of CaOx formed in the original urine sample from the COM standard curve (real iCOCI absorbance divided by slope of standard curve), and the obtained value was called urinary iCOCI value. The urinary iCOCI values were expressed as COM equivalent in g/L or g/day or g/g creatinine (COM eqv., g/L or g/day or g/g Cr). The prototype of urinary iCOCI test kit was available in our lab (patent pending). The iCOCI measurements were done in urine samples in a random way, interspersed between urine samples of villagers and hospitalized subjects.

To optimize the incubation time required for CaOx crystallization after adding CaCl_2_, we performed iCOCI measurement in pooled urine samples from SFS and NSS compared among seven different incubation time points, 0 (immediately after adding CaCl_2_), 10, 20, 30, 40, 50 and 60 min. We found no significant difference in iCOCI values among them neither in SFS nor NSF pooled urine samples (Supplementary Fig. 2). This suggests that complete crystallization of CaOx rapidly occurred. We, therefore, chose the incubation time of 10 min in order to shorten the iCOCI procedure.

We also tested whether indole reaction was selective to oxalate. Substances commonly found in urine, including urea, creatinine, phosphate, UA, citrate and lactate (three concentrations for each) were tested for reactivity with indole reagent. We found that all tested substances had no or very little reactivity with indole reagent, except UA (Supplementary Fig. 3). UA showed some detectable reactivity with indole reagent at 1 mM (168 mg/L), but the yielded color (brownish) was not the same with the color produced from oxalic acid (pinkish). All tested oxalate-containing substances (sodium oxalate, COM and oxalic acid) showed well selective reactivity with indole reagent and generated the same pinkish product (Supplementary Fig. 3). According to FTIR result of our previous report^11^, we did not see UA crystals in the COCI crystals harvested from the COCI procedure. Therefore, we were confident that the pink-colored compound yielded in the iCOCI procedure was the product specifically derived from oxalate existed in the COCI crystals.

### Statistical analysis

Data were presented as mean ± standard deviation (SD) or median (interquartile range, IQR), as appropriated. Differences in urinary iCOCI levels between NSS and SFS were tested by two-sample t-test and Mann Whitney test. ROC analysis was performed to assess how well the urinary iCOCI test was able to distinguish SFS from NSS. Cutoff value was chosen based on an intention to use the urinary iCOCI test as a screening test with high sensitivity and high negative predictive value (NPV). Diagnostic values including sensitivity, specificity, positive predictive value (PPV), NPV, false positive rate (FPR), false negative rate (FNR) and accuracy were calculated regarding to each selected cutoff. Positive likelihood ratio (LH^+^) was calculated from sensitivity/(1-specificity), and negative likelihood ratio (LH^−^) was calculated from (1-sensitivity)/specificity^21^. Logistic regression was carried out to calculate the adjusted odds ratio (OR) of positive result of the urinary iCOCI test. GraphPad Prism 6.0 and Stata version 12 were employed for all statistical calculations. P value < 0.05 was considered as statistically significant.

## Results

We designed and recruited participants in accordance with the STARD 2015 statement^14^. Urinary iCOCI test was an evaluated index test. CT scan was a reference standard. Urinary iCOCI values were determined in 24-h urine samples obtained from the 281 recruited subjects (Fig. 1). CT scan was subsequently performed as a main imaging modality to diagnose the urinary stone condition. The subjects were divided into two groups NSS (n = 122) and SFS (n = 159). Mean age of NSS and SFS were 50.68 ± 9.43 and 56.66 ± 10.72 years old, respectively. Urine volume between the two groups were comparable (1.33 ± 0.64 vs. 1.42 ± 0.60 L, P > 0.05) (Table 1).

**Figure 2:**
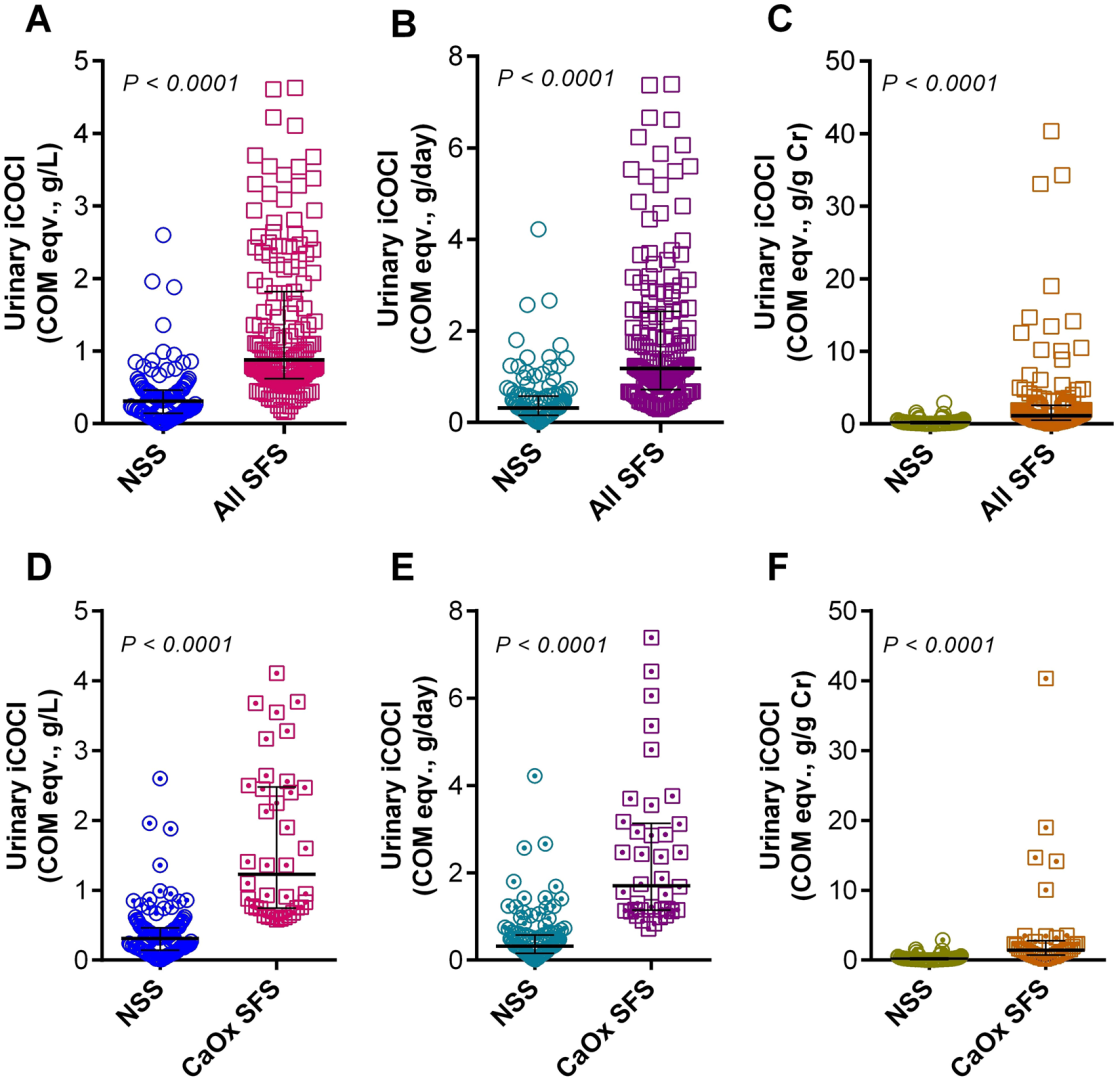
Urinary iCOCI levels compared between NSS and all SFS (**A–C**), and between NSS and CaOx SFS (**D–E**). Urinary iCOCI values in stone groups (both all stone and only CaOx stone) were significantly higher than non-stone control group (for all three units). Error bars indicate median and IQR.

Urinary iCOCI levels in SFS were significantly higher than NSS either expressed in COM eqv., g/L (1.31 ± 1.01 vs. 0.37 ± 0.38, P < 0.001), COM eqv., g/day (1.82 ± 1.61 vs. 0.48 ± 0.58, P < 0.001) or COM eqv., g/g Cr (2.73 ± 5.50 vs. 0.27 ± 0.37, P < 0.001) (Fig. 2). Of 159 SFS, 16 were asymptomatic stone subjects from the villages, and 143 were symptomatic stone subjects from the hospital. Urinary iCOCI levels between villager SFS (1.03 ± 0.46 COM eqv., g/L) and hospital SFS (1.34 ± 1.05 COM eqv., g/L) were not significantly different (P = 0.341), but they were significantly higher than that in NSS controls (P < 0.001). Notably, all 16 asymptomatic SFS from the villages were positive for urinary iCOCI test (> 0.6 COM eqv., g/L).

**Figure 3:**
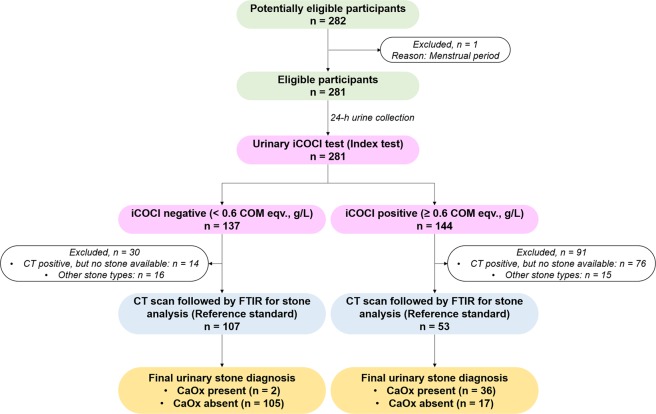
STARD flow diagram of the study for CaOx stone cases (n = 38).

Similar to all SFS, urinary iCOCI levels in CaOx SFS were significantly greater than the NSS either expressed in COM eqv., g/L (1.65 ± 1.09 vs. 0.37 ± 0.38, P < 0.001), COM eqv., g/day (2.41 ± 1.71 vs. 0.48 ± 0.58, P < 0.001) or COM eqv., g/g Cr (3.86 ± 7.43 vs. 0.27 ± 0.37, P < 0.001) (Fig. 2). The STARD flow diagram for CaOx SFS is shown in Fig. 3.

**Figure 4:**
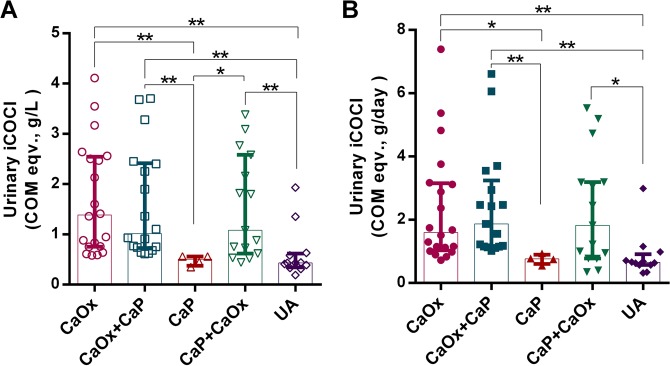
Urinary iCOCI levels compared among SFS with different stone types (n = 69). On average, urinary iCOCI in CaOx SFS (1.71 ± 1.11 COM eqv., g/L, n = 20) was higher than CaOx + CaP (1.60 ± 1.10 COM eqv., g/L, N = 15), CaP + CaOx (1.54 ± 1.03 COM eqv., g/L, n = 15), UA (0.62 ± 0.51 COM eqv., g/L, n = 12) and CaP (0.48 ± 0.10 COM eqv., g/L, n = 4), respectively. *P < 0.05, **P < 0.001. Error bars indicate median and IQR.

Of 159 SFS, 69 cases had stone specimens available for mineral composition analysis. According to FTIR spectra (Supplementary Fig. 1), 69 stones were categorized into 5 groups according to the main mineral composition, *i.e*., CaOx (n = 20), CaOx + CaP (n = 18), CaP (n = 4), CaP + CaOx (n = 15) and UA (n = 12). Urinary iCOCI levels in CaOx (1.71 ± 1.11 COM eqv., g/L) and CaOx + CaP (1.60 ± 1.10 COM eqv., g/L) groups were significantly higher than in CaP (0.48 ± 0.10 COM eqv., g/L) and UA (0.62 ± 0.51 COM eqv., g/L) groups (Fig. 4). Likewise, urinary iCOCI levels in CaP + CaOx (1.54 ± 1.03 COM eqv., g/L) group were significantly greater than in CaP and UA groups.

**Figure 5:**
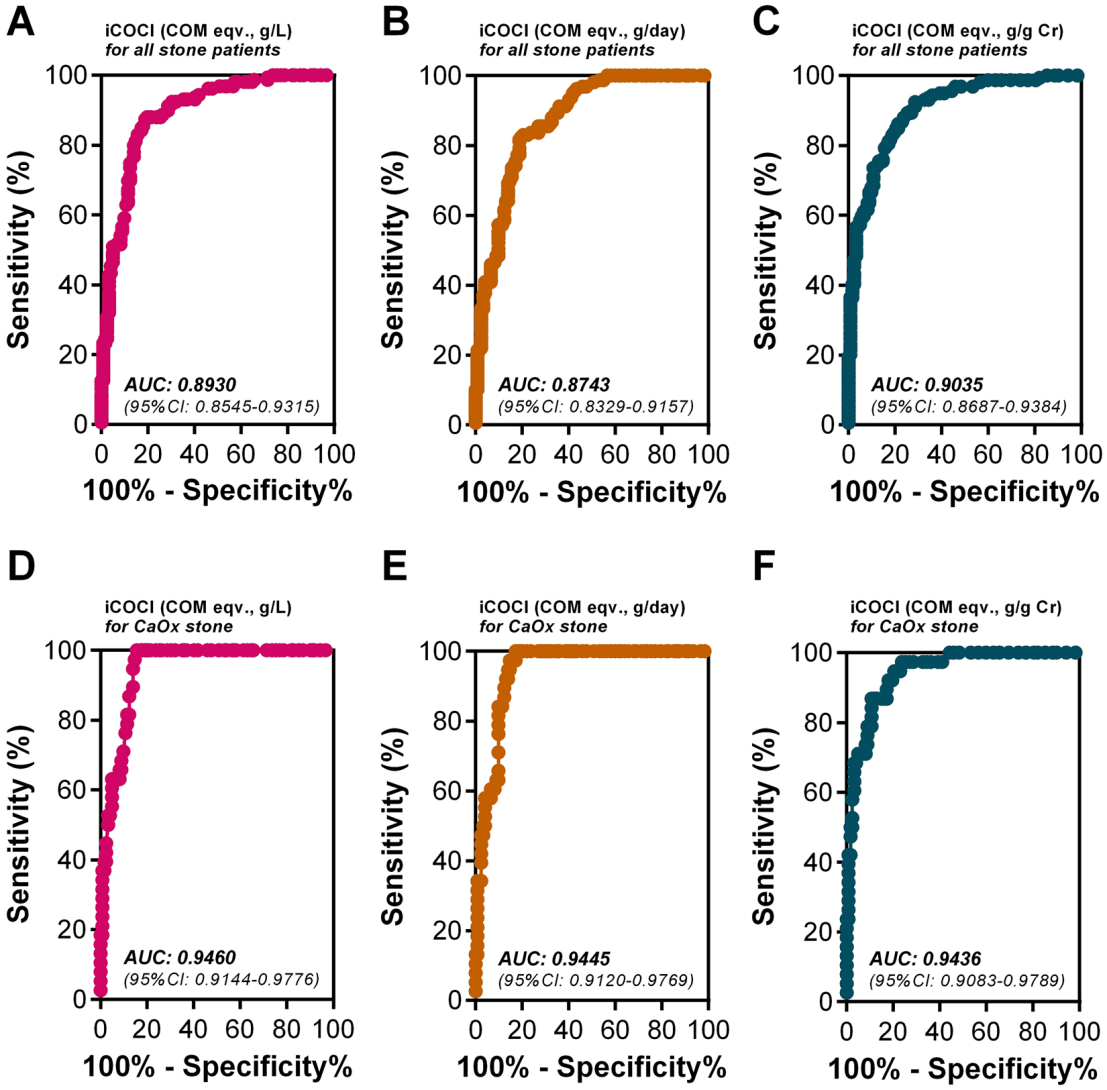
ROC curves to assess overall performance of urinary iCOCI test in separating non-stone from stone conditions (**A–C** from all stone cases, **D–F** from CaOx stone cases). The results show that the urinary iCOCI test had an excellent accuracy for distinguishing between the NSS and CaOx SFS, as the AUC were over 0.9. Urinary iCOCI expressed in COM eqv, g/L had the highest AUC (**D**) relative to the other units (**E,F**), indicating the best diagnostic performance.

According to the ROC analysis results, the diagnostic performance of urinary iCOCI test in separating NSS from all SFS was lower (indicated by a lower AUC) than separating NSS from CaOx SFS (Fig. 5). In separating NSS from CaOx SFS, urinary iCOCI expressed in COM eqv., g/L (0.9460, 95% CI: 0.9144–0.9776) had higher AUC than urinary iCOCI expressed in COM eqv., g/day (0.9445, 95% CI: 0.9120–0.9769) and in COM eqv., g/g Cr (0.9436, 95% CI: 0.9083–0.9789), respectively (Fig. 5). These suggest that urinary iCOCI test expressed in COM eqv., g/L has the highest power for urolithiasis diagnosis. According to the ROC guideline^22,23^, it can be concluded that urinary ICOCI test is excellently accurate (AUC between 0.9 and 1) in separating NSS from CaOx SFS. However, urinary iCOCI test has a bit lower accuracy in discriminating NSS from all SFS.

Cutoff values of urinary iCOCI test that gave the highest sensitivity and accuracy were selected. The selected cutoffs were 0.6 COM eqv., g/L, 0.8 COM eqv., g/day and 0.3 COM eqv., g/g Cr. The diagnostic values of each cutoff are shown in Table 2. Sensitivity, specificity, PPV, NPV, FPR, FNR, accuracy, LH^+^ and LH^−^ of urinary iCOCI test (expressed in COM eqv., g/L) for diagnosing CaOx urolithiasis were 95%, 86%, 68%, 98%, 32%, 2%, 88%, 6.80 and 0.06, respectively (Table 2).

**Table 2:** Diagnostic values of urinary iCOCI test in distinguishing NSS form all SFS and only CaOx SFS.

	Urinary iCOCI (COM eqv. g/L)	Urinary iCOCI (COM eqv. g/day)	Urinary iCOCI (COM eqv. g/g Cr)
Cutoff values	0.6	0.8	0.3
For all stone cases			
• Sensitivity	87.25	70.44	89.31
• Specificity	80.33	85.25	74.59
• PPV	84.42	86.15	82.08
• NPV	83.76	68.87	84.26
• FPR	15.58	13.85	17.92
• FNR	16.24	31.13	15.74
• Accuracy	82.56	76.87	82.92
• LH^+^	4.44	4.78	3.51
• LH^−^	0.16	0.35	0.14
For CaOx stone cases			
• Sensitivity	94.74	97.37	97.37
• Specificity	86.07	85.25	74.59
• PPV	67.92	67.27	54.41
• NPV	98.13	99.05	98.91
• FPR	32.08	32.73	45.59
• FNR	1.87	0.95	1.09
• Accuracy	88.13	88.13	80.00
• LH^+^	6.80	6.60	3.83
• LH^−^	0.06	0.03	0.04

PPV: positive predictive values, NPV: negative predictive value, FPR: false positive rate, FNR: false negative rate, LH^+^: positive likelihood ratio, LH^−^: negative likelihood ratio.

Logistic regression was performed to find the strength of association of positive urinary iCOCI test (≥ 0.6 COM eqv., g/L) with urolithiasis condition. After adjusted for age, sex, BMI, urine volume and urine pH, the adjusted OR of positive urinary iCOCI was 21.39 (95% CI: 10.40–44.00), meaning that individuals with positive result had about 21 times higher risk for urolithiasis than those with negative results. For CaOx urolithiasis, adjusted OR of positive urinary iCOCI was 277.67 (95% CI: 27.82–2770.97), indicating that individuals with positive result had about 278 times higher risk for CaOx stone formation than those with negative result.

## Discussion

Urinary stone disease is a long-standing urologic problem happened to mankind. However, an innovative test with high accuracy, simple procedure and cost-effectiveness for assessing the likelihood of having or not having urolithiasis has not been established for the routine use. Several risk index for CaOx stone formation had been established, and their clinical accuracy have been evaluated. The most widely accepted stone risk indexes are RS_CaOx_ (in 1985), AP_CaOx_ (in 1997) and BRI (in 2000)^10,24^. In 2014, we developed a new urinary COCI test for quantifying the total CaOx crystallization capacity in urine samples^11^. Although both BRI and COCI tests were reported with high diagnostic power, these studies were case-control retrospective studies, and the determination of BRI and COCI were performed in the post-diagnostic urine specimens. These could harbor bias, in particular the spectrum bias^20^. In this study, we improved the COCI procedure to selectively measure only CaOx crystals by combining the original COCI procedure with an indole-oxalate reaction. The clinical validation of urinary iCOCI test was tested by complying with the STARD guideline^14^. To minimize the spectrum bias, the urinary iCOCI test was performed in the 24-h urine samples taken before diagnosis.

Diagnostic confirmation of urolithiasis usually requires imaging modalities, and the most commonly used imaging tools are ultrasonography (US), KUB plain film radiography and CT scan^25^. Non-contrast CT scan has been considered as a gold-standard method for detecting urinary stones with high sensitivity, specificity and accuracy^25,26^. Sensitivity of US, KUB X-ray and CT scan for stone diagnosis have been reported at 45%, 58–62% and 95–100%, respectively^27^. Although the CT scan is highly accurate, radiation exposure is the main concern (approximately 14 times higher than KUB X-ray)^25^. Also, it is costly relative to other imaging modalities (10 times higher than KUB X-ray, 2 time higher than US). Therefore, to reduce the number of suspicious subjects requiring CT scan for confirming the presence of urinary stones, an effective non-invasive screening test must be developed. We demonstrated herein the clinical utility of urinary iCOCI test in screening CaOx urolithiasis.

Levels of urinary iCOCI in SFS were significantly higher than NSS. Among three units of urinary iCOCI, unit of COM eqv., g/L provided the best diagnostic power. We recommended to use this unit for the clinical implication. For separation of NSS from all stone cases, AUC of 0.893 (95% CI: 0.855–0.932) was obtained, indicating that the test was moderately accurate (AUC between 0.7 and 0.9)^22,23^. When considered only CaOx stone cases (excluded all other stone types), the discriminatory power of urinary iCOCI test in distinguishing CaOx SFS from NSS was remarkably increased (AUC: 0.946, 95% CI: 0.914–0.978) with higher sensitivity (94.74%) and NPV (98.13%). Additionally, FNR was found only 1.87%. This indicated that urinary iCOCI test was efficient and highly accurate for screening CaOx calculi.

Ideally, screening test would be positive only in individuals who have the disease and negative in those do not have the disease, indicating the perfect (100%) diagnostic accuracy. Most of the actual screening tests, however, exhibit some levels of false positive and false negative rates^19^. Predictive values (PPV and NPV) of the test are clinically useful, but these values depend on the disease prevalence and frequently varies among studies^28^. Likelihood ratios are alternative statistics to summarize the diagnostic accuracy, predict the risk of disease and be clinically helpful to rule in or rule out the disease regardless of the disease prevalence^21^. It is well accepted that LH^+^ of 10 or more and LH^−^ of 0.1 or less represent an excellent informative screening test^29^. In this study, we obtained the LH^−^ of 0.06 for predicting CaOx urolithiasis. This means that individual with a negative result of urinary iCOCI test has 17-fold (1/0.06) decrease in the odds of developing CaOx calculi. Therefore, urinary iCOCI test was clinically useful for ruling out the disease, in this case CaOx urolithiasis. It is reasonable to implement this urinary iCOCI test as a screening test in the clinical settings.

The numeric value of the urinary iCOCI test reflected amount of CaOx crystals precipitated in the urine sample under a specific condition. It was calculated by subtraction from amount of CaOx formed in the 2 mM oxalic acid solution. Since we used COM solutions to create the calibration curve, the unit of urinary iCOCI was expressed as COM equivalent. It should be noted that this iCOCI unit was arbitrary unit, and it did not relate to the actual level of oxalate in the urine sample. In other word, concentration of urinary oxalate could not be directly calculated from the urinary iCOCI value. For example, at the urinary iCOCI of 1.82 COM eqv. g/d, the urinary oxalate excretion directly derived from this value would be 1.096 g/d (COM molecular weight: 146.11 g/mol, oxalate molecular weight: 88.02 g/mol). This derived oxalate level is extremely high and not clinically possible (24-fold higher than normal), as the normal urinary oxalate excretion is less than 0.5 mmol/d or 45 mg/d. Even at our selected cutoff (0.8 COM eqv. g/d), it would be corresponded to the urinary oxalate of 482 mg/d that was 11-time higher than normal. Therefore, the actual urinary oxalate should not be directly calculated from the urinary iCOCI value. This raises a question: what does the iCOCI test actually measure? Whether this irrelevance is due to the non-specific reaction of indole reagent or the incorrect subtraction of 2 mM spiked oxalic acid. In our previous report of urinary COCI test^11^, we analyzed the mineral composition of the harvested COCI crystals by FTIR, and we found no UA crystals (mainly were CaOx and CaP). Although UA was found to be positive for indole reaction (but much lesser extent than oxalate) (Supplementary Fig. 3), it was unlikely that UA had a significant impact on the iCOCI value because there were no UA crystals detected in the COCI crystals. Nevertheless, it might be possible that minute amount of UA formed in the COCI crystals (undetectable by FTIR) slightly contribute to the increase in iCOCI value.

As mentioned in the Method section, the 2 mM oxalic acid solution used for correcting iCOCI absorbance (to obtain the real iCOCI absorbance in original urine sample) was prepared in distilled water. Thus, CaOx crystallization of this 2 mM oxalic acid solution after adding CaCl_2_ was merely occurred in water. In contrast, CaOx crystallization of the 2 mM spiked oxalic acid (in urine sample) was occurred in urine. Different matrix influences differently on the CaOx crystal formation. Urine contains a lot of inorganic and organic substances, and these matrices, particularly cellular biomolecules, have been shown to have influence on CaOx formation. Albumin purified from urine promoted CaOx crystallization^30^. Osteopontin enhanced formation and aggregation of CaOx crystals^31^. Our previous study also demonstrated that urinary lipids, especially glycolipids and cholesterol, promoted CaOx crystallization and aggregation^32^. These suggest that cellular biomolecules in urine augment the CaOx crystal formation. It might be possible that amount of CaOx crystals yielded in urine was greater than that yielded in water, hence causing increase in iCOCI value. However, these are our speculations, and further experimental proof is required. Mechanistic insight into chemistry of CaOx crystallization in the COCI procedure and indole reaction are under investigation.

Limitations of the study should be mentioned. Although the study sites were in the stone endemic area, it was only in one province, Mahasarakham province. Therefore, the studied subjects might not be a perfect representative of the whole country. In this study, we did not consider the subject, who was stone former in the past, but had no stone detected by CT scan at the time of recruitment, as SFS. Only subjects who currently had urinary stone (positively detected by CT scan, regardless of stone sizes) at the time of study were classified as SFS. Sex distribution between SFS and NSS groups was not perfectly equal. It might confound some minor findings, but we believed that the main conclusive findings remained accurate.

Conclusion, the urinary iCOCI test was invented. We noted that the value of urinary iCOCI was calculated and expressed as an arbitrary unit reflecting amount of CaOx formed in the conditioned urine. The test measured the total capability of urine that was spiked with 2 mM oxalic acid to precipitate out CaOx crystals under the excessive calcium condition rather than directly measured the actual level of oxalate in urine. Therefore, urinary oxalate level should not be directly derived from the urinary iCOCI value. We clinically validated the diagnostic accuracy of this innovative urinary iCOCI test for urolithiasis, and found that the test had excellent discriminatory power to distinguish NSS from CaOx SFS with high sensitivity (95% for CaOx stone, 87% for all stones) and high NPV (98% for CaOx stone, 84% for all stones). Significantly low likelihood ratios for negative test results (LH^−^ : 0.06 for CaOx stone, 0.16 for all stones) were obtained. These diagnostic data suggest that the urinary iCOCI test is highly accurate and clinically useful. It can be implemented as a screening test to determine the probability of urolithiasis development, especially CaOx calculi, in the high-risk population. In individuals with positive urinary iCOCI test, but have no symptom related to stone disease, we suggest giving them advice on lifestyle modification, particularly changing dietary habit, to be more protective for urinary stone formation such as increased fluid intake, restriction of high-oxalate diets and increased consumption of citrus fruits and juices. In contrast, negative result of urinary iCOCI test is highly accurate to rule out the presence of CaOx calculi.

## Supplementary information

Supplementary figures.
