# Supplementary material for: Clinical validation of urinary indole-reacted calcium oxalate crystallization index (iCOCI) test for diagnosing calcium oxalate urolithiasis
Source: Sci Rep. 2020 May 20;10:8334. doi: 10.1038/s41598-020-65244-1 (PMC7239845; doi:10.1038/s41598-020-65244-1)

**Title page**

**Type of manuscript:** Original article

**Title:** Clinical validation of urinary indole-reacted calcium oxalate crystallization index (iCOCI) test for diagnosing calcium oxalate urolithiasis

**Authors**

Pimkanya More-krong^1^, Praween Tubsaeng^2^, Natcha Madared^1^, Monpichar Srisa-Art^3^, Numpon Insin^3^, Pannee Leeladee^3^, Chanchai Boonla^1,*^

**Affiliations**

^1^Department of Biochemistry, Faculty of Medicine, Chulalongkorn University, Bangkok 10330 Thailand

^2^Division of Urology, Mahasarakham Hospital, Mahasarakham Province 44000 Thailand

^3^Department of Chemistry, Faculty of Science, Chulalongkorn University, Bangkok 10330 Thailand

**Running title:** urinary iCOCI as a screening test for calcium oxalate urolithiasis

**Corresponding author**

Chanchai Boonla, Ph.D., Assistant Professor

Department of Biochemistry, Faculty of Medicine

Chulalongkorn University, Bangkok 10330 Thailand

Tel/Fax: +66-2-2564482

Email: [chanchai.b@chula.ac.th](mailto:chanchai.b@chula.ac.th)

**Supplementary Fig. 1** Representative FTIR spectra of each stone type, i.e., CaOx (A), CaOx+CaP (B), CaP (C), CaP+CaOx (D) and UA (E) stones.

**
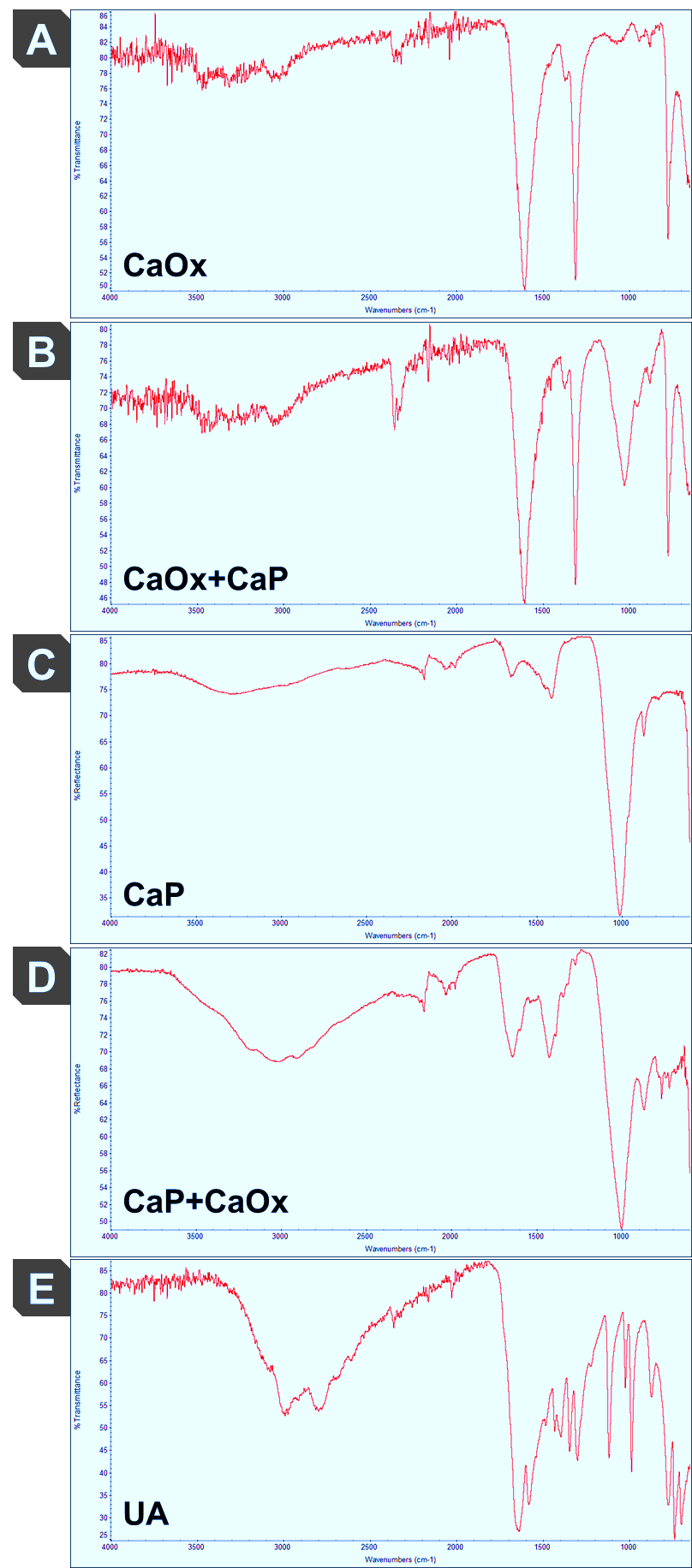
**

**Supplementary Fig. 2** Comparison of urinary iCOCI values of SFS and NSS urine samples measured at different incubation times after addition of CaCl_2_ solution. No significant difference in urinary iCOCI values among the different incubation time periods was found neither in SFS nor NSF urine samples.


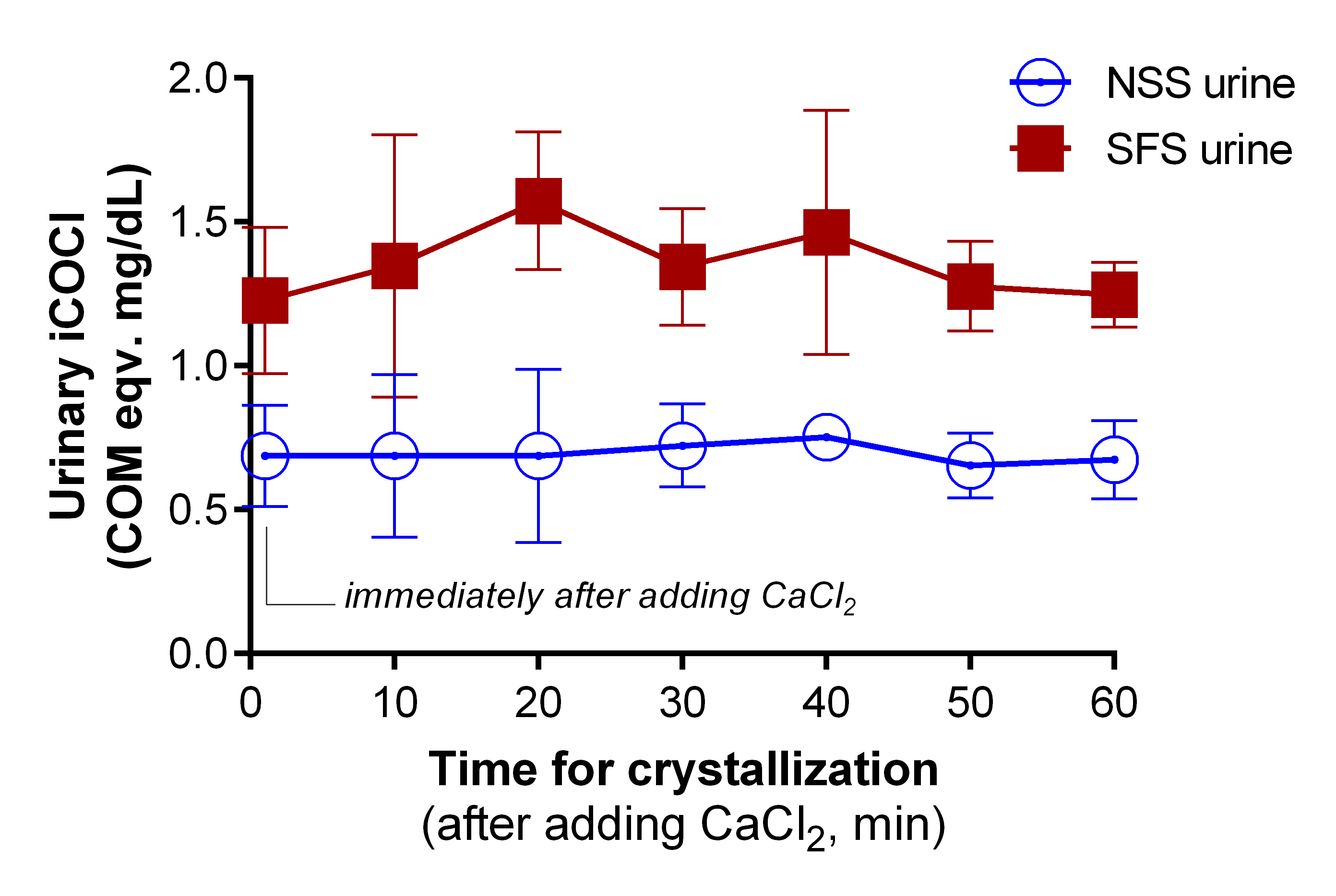


**Supplementary Fig. 3** Testing of selectivity of indole reaction to oxalate. Three varied concentrations of urea, creatinine, phosphate, UA, citrate and lactate were tested for reactivity with indole reagent. Regarding to absorbance at 530 nm, no substances reacted with indole reagent, except UA (A). However, the color generated by UA (brownish) was not the same with that yielded by oxalate (pinkish) (B). Sodium oxalate, COM and oxalic acid selectively reacted with indole reagent generating the pink-colored product. The results clearly demonstrated that indole reaction is very well selective to oxalic acid.


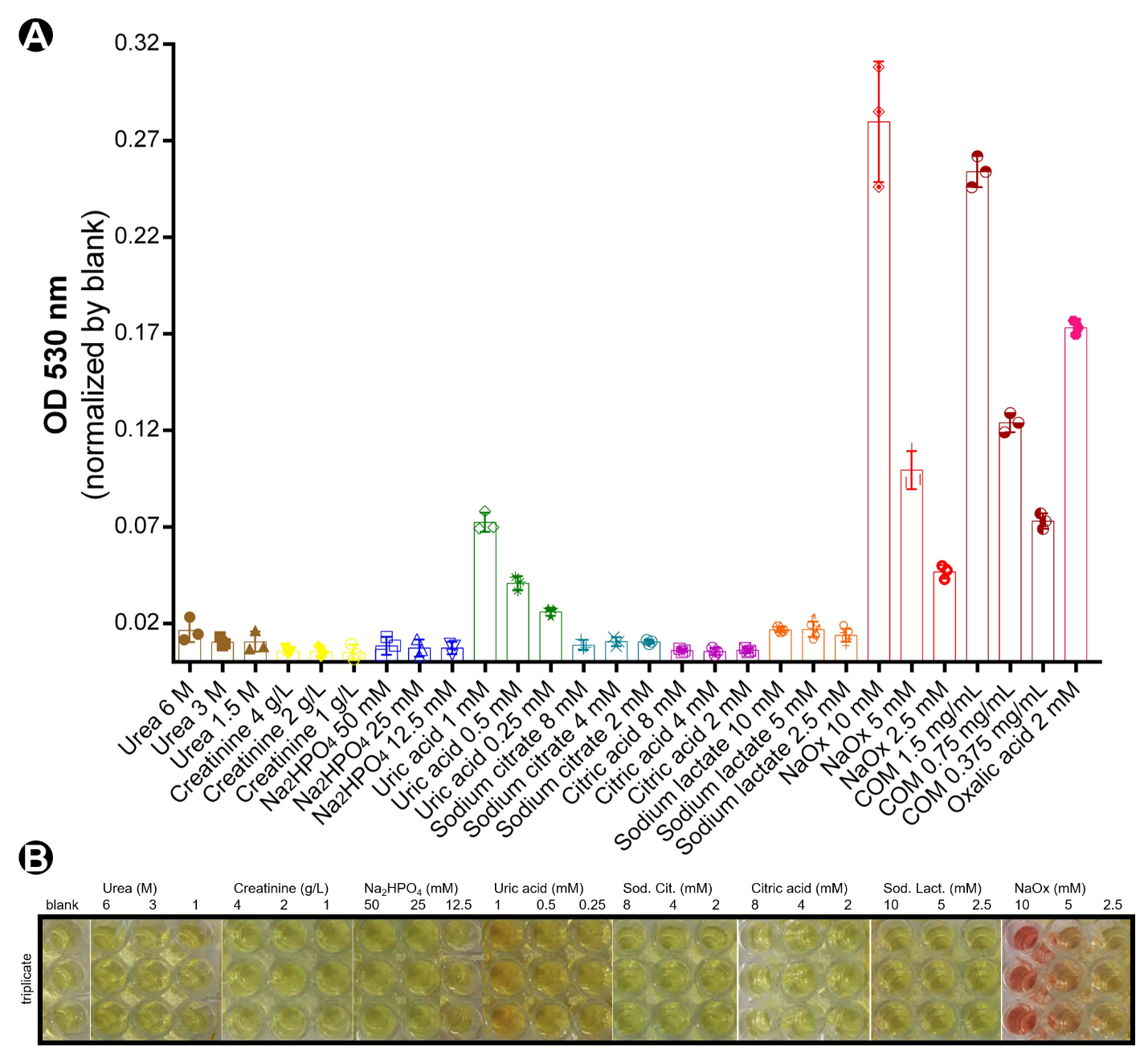

Supplement: Supplementary file 1 — Supplementary figures. [file 41598_2020_65244_MOESM1_ESM.doc]
